# Meta-Transcriptomic Response to Copper Corrosion in Drinking Water Biofilms

**DOI:** 10.3390/microorganisms13071528

**Published:** 2025-06-30

**Authors:** Jingrang Lu, Ian Struewing, Nicholas J. Ashbolt

**Affiliations:** 1 U.S. EPA Office of Research and Development, Cincinnati, OH 45268, USA; struewing.ian@epa.gov; 2 Future Industries Institute, University of South Australia, Adelaide, SA 5005, Australia; nicholas.ashbolt@unisa.edu.au

**Keywords:** biofilm, transcriptomic analysis, copper, PVC, microbial organism, drinking water, tubing

## Abstract

Drinking water biofilm ecosystems harbor complex and dynamic prokaryotic and eukaryotic microbial communities. However, little is known about the impact of copper corrosion on microbial community functions in metabolisms and resistance. This study was conducted to evaluate the impact of upstream Cu pipe materials on downstream viable community structures, pathogen populations, and metatranscriptomic responses of the microbial communities in drinking water biofilms. Randomly transcribed cDNA was generated and sequenced from downstream biofilm samples of either unplasticized polyvinylchloride (PVC) or Cu coupons. Diverse viable microbial organisms with enriched pathogen-like organisms and opportunistic pathogens were active in those biofilm samples. Cu-influenced tubing biofilms had a greater upregulation of genes associated with potassium (K) metabolic pathways (i.e., K-homeostasis, K-transporting ATPase, and transcriptional attenuator), and a major component of the cell wall of mycobacteria (mycolic acids) compared to tubing biofilms downstream of PVC. Other upregulated genes on Cu influenced biofilms included those associated with stress responses (various oxidative resistance genes), biofilm formation, and resistance to toxic compounds. Downregulated genes included those associated with membrane proteins responsible for ion interactions with potassium; respiration–electron-donating reactions; RNA metabolism in eukaryotes; nitrogen metabolism; virulence, disease, and defense; and antibiotic resistance genes. When combined with our previous identification of biofilm community differences, our studies reveal how microbial biofilms adapt to Cu plumbing conditions by fine-tuning gene expression, altering metabolic pathways, and optimizing their structural organization. This study offers new insights into how copper pipe materials affect the development and composition of biofilms in premise plumbing. Specifically, it highlights copper’s role in inhibiting the growth of many microbes while also contributing to the resistance of some microbes within the drinking water biofilm community.

## 1. Introduction

Premise plumbing (PP) biofilms are diverse microbial ecosystems that can harbor waterborne pathogens, which are dependent upon pipe materials and other local niche conditions. Microbial activity in the biofilms may cause adverse effects in PP, including pipe corrosion, water color problems, water taste and odor, disinfectant residuals, and the spread of waterborne pathogens [1,2,3]. Although the survival and regrowth of drinking water microorganisms in PP can be affected by many factors, the effects of pipe materials on biofilm formation and regrowth are known to be quite complex [4]. Copper (Cu), a widely used pipe material in drinking water premise plumbing systems, has long been considered to suppress microbial growth in drinking water [5] and, therefore, likely influences the growth of microbial pathogens. Biofilms growing on Cu materials have lower biomass and diversity compared to biofilms growing on other common PP materials, such as polyvinyl chloride (PVC) [6,7,8,9]. Cu-based biofilms also have been shown to have slower biofilm growth rates with different community structures [10,11]. Notably, the Cu pipe biofilms lowered the microbial diversity in downstream biofilms on tubing materials [12].

Contrary to the suppressive effects of Cu on biofilms, generally, our team reported preferential colonization and the release of *Legionella pneumophila* on Cu-associated biofilms compared to those from PVC. Additionally, this strain exhibited increased persistence in warm water alongside the free-living *Legionella* host, *Vermamoeba vermiformis* [13,14,15]. An examination of Cu versus PVC biofilm microbiomes in more detail showed an increase in the relative abundance or dominance of some Cu-resistant microbes like *Mycobacterium* spp. and free-living amoebae [12]. However, these previous studies were based on DNA analysis, which may not reflect a viable/active community, the mechanisms of impact, nor community functions under the apparent Cu effect. When others examined rDNA and rRNA sequences from biofilms to identify active members, mixed results were observed but they generally indicated less mycobacteria in Cu biofilms than those from silane cross-linked polyethylene [16]. What appears unreported, however, are the upstream Cu materials’ impacts on downstream microbiomes grown on different pipe materials.

Considering that both Cu and PVC pipe materials are commonly used in jointed pipelines for premise water systems, this study was designed to investigate the impact of corrosion from Cu versus PVC pipes on downstream biofilms. The aim of this study was to evaluate the impact of upstream Cu pipe materials against PVC pipe materials on downstream biofilms colonizing a plastic tubing (a third material) in terms of viable community structures, pathogen populations, and metatranscriptomic responses. This study provides additional insights into the potential impacts of released Cu on biofilm organisms/metabolites.

## 2. Materials and Methods

### 2.1. Experiment and Sample Collection

The design of this experiment, sampling procedures, and water quality parameters (temperatures, free and total chlorine, pH, conductivity, and dissolved oxygen) were reported previously [11,12]. The microbial community compositions of the source, i.e., City of Cincinnati drinking water, have also been described [17,18,19]. Sample collection for this study was designed to study biofilms in the tubing effluent lines of CDC bioreactors, which was meant to represent downstream PP like that in a shower head or similar such household fixture [20]. Figure S1A illustrates the setup of one of the four bioreactors: two containing Cu coupons and the other two with PVC coupons. The bioreactors were fed with ambient (20.8 ± 1.6 °C) Cincinnati tap water that was held in a light-protected storage tank (23 L) to allow for natural dechlorination (free chlorine residual from the tank: 0.169 ± 0.175 mg L^−1^), which is expected in distal PP. Each was fed tap water at 40 mL h^−1^ (10 h hydraulic resident time) using a peristaltic pump (Cole-Parmer, Vernon Hills, IL, USA) (Figure S1B). To simulate the last leg of a PP system, the CDC bioreactor effluent line consisted of Norprene^TM^ food-grade tubing with 3/8″ inner diameter (Cole-Parmer) (Figure S1A). In-premise stagnation/disturbance periods were simulated by placing bioreactors on a magnetic stir plate that was activated every 2 h for 30 min at 100 rpm (Figure S1C). Each reactor contained 8 coupon holders, and each bioreactor vessel contained 24 coupons with a surface area of 1.27 cm^2^ Cu or PVC coupon-1 (Figure S1D). After one year of biofilm incubation on the coupons within bioreactors, the effluent lines were replaced with new Norprene^TM^ tubing. The tubing biofilm grew for 140 days and the Norprene^TM^ biofilm samples were collected by the end of incubation. After stopping the bioreactor system and detaching the Norprene^TM^ tubing from the bioreactor outlet, a 60–90 cm segment of the tubing from each bioreactor was sampled by cutting it longitudinally and collecting the colonized biofilm aseptically with a sterilized stainless-steel scraper. Approximately 13 cm^2^ of the tubing surface of each effluent line was sampled. The sampled biofilm with wet weight biomass (0.3–0.7 g or 23–53 mg cm^−2^) was placed into a pre-weighted bead beating tube with 0.1 mm silica beads (MP Biomedicals LLC, Solon, OH, USA) and 300 µL T&C lysis buffer (Epicenter Biotechnologies, Madison, WI, USA). The bead beating tubes were placed under −80 °C for the following RNA isolation. For each of the two bioreactor replicates, there were four replicates of biofilm samples, as replicates for each effluent line or bioreactor were used in this study. For the sequencing purpose, the total of 8 biofilm samples were treated as biological replicates. The PVC and Cu bioreactor tubing biofilms are denoted as PVC- and Cu-influenced biofilms, respectively, hereinafter.

### 2.2. Total Biofilm RNA Isolation

Total RNA isolation was carried out shortly after the collection of samples using TRIzol^®^ Reagent (Life Technology, Carlshad, CA, USA). Briefly, the replicate frozen biofilm samples were quickly thawed and then disrupted and lysed using a Mini-Beadbeater-16 (BioSpec Products, Inc., Bartlesville, OK, USA) bead-beating twice for 30 s and then centrifuged at 10,000× *g* for 3 min. The supernatant was then transferred to a new sterile tube, and RNA was extracted following the manufacturer’s instructions. The extracted RNA was dissolved in 20 µL RNase-free water (Sigma-Aldrich, St. Louis, MO, USA) and subsequently treated to remove genomic DNA using the TURBO DNA-free™ Kit (Life Technologies, Foster City, CA, USA). The RNA quality was assessed electrophoretically on an Agilent 2100 bioanalyzer (Agilent Tech. Inc., Santa Clara, CA, USA). RNA quantity was estimated using a Nanodrop ND-1000 spectrophotometer (NanoDrop Technologies, Inc., Wilmington, DE, USA) and then stored at −80 °C until used.

### 2.3. cDNA Synthesis, Library Construction, and Sequencing

Approximately 200 ng of each resultant RNA was treated with Ribo-Zero (Illumina, San Diego, CA, USA) to remove rRNA and then purified using RNeasy Mini RNA™ isolation kit (Qiagen, Hilden, Germany). The size spectra of resultant mRNA were around 200–400 bp; thus, a fragmentation step was omitted. The first- and second-strand cDNA were synthesized using the Superscript™ III First-Strand Synthesis SuperMix (Life Technology, Carlsbad, CA, USA) and the SuperScript™ Double-Stranded cDNA Synthesis Kit (Life technologies, Carlsbad, CA, USA), respectively, following manufacturer’s instructions. The resultant cDNA was purified and cDNA libraries were prepared following the manufacturer’s protocol using an Illumina NexteraXT DNA library prep kit (Illumina, San Diego, CA, USA). The quality and size spectra of cDNA libraries were examined using a Bioanalyzer (Agilent 2100, Agilent Tech. Inc., Palo Alto, CA, USA). Sequencing was performed and data were generated from paired end reads (2 × 300 bp) on a MiSeq system (Illumina, San Diego, CA, USA). The image analysis and base calling were performed using the Illumina pipeline with default settings. Raw reads with >10% unknown nucleotides or with >50% low-quality nucleotides (quality value < 20) were discarded [21]. Three sequencing depths, i.e., 0.5 Gbp, 1.0 Gbp, and 2.4 Gbp reads, were applied for both metatranscriptomic datasets.

### 2.4. Sequence Data Analyses

The taxonomic classification was performed based on sequence reads derived from cDNA. Taxonomic domain information analysis was conducted using the MG-RAST (Meta Genome Rapid Annotation using Subsystem Technology, v3.3) server at the Argonne National Library (http://metagenomics.anl.gov, accessed on 16 September 2014), which allows for the retrieval of sequences from several databases, including NCBI-nr, IMG, KEGG, COG, SEED, Subsystems, etc., performing a single similarity search on this server [22]. In the present study, the tubing biofilm RNA dataset (MG-RAST ID: mgp10718 from 4580456.3 to 4580491.3) was analyzed mostly using Subsystems database [22].

### 2.5. Global Gene Expression Classification

To obtain whole metabolic pathway information, global gene expression was annotated with SEED Subsystems in MG-RAST. The RNA-Seq dataset stored in the MG-RAST with a total of 2.4 Gbp cDNA was analyzed primarily using the Subsystems database, which set the cut-off values at a maximum e-value of 1 × 10^−5^, a minimum identity of 60%, and a minimum alignment length of 15 measured in amino acids for protein and base pairs for RNA databases [22].

The MG-RAST v3 annotation pipeline uses the steps to map one read to multiple annotations and one annotation to multiple reads through three steps: not-one-to-one gene prediction step, clustering identified sequences at 90% amino acid identity, and performing one search for each cluster and the not-one-to-one annotation process itself [22]. Relative abundance (RA: %) was calculated as the percentage of the retrieved sequence numbers for each category divided by total sequences in each library. To identify the main metabolic categories involved in various specific metabolisms, the number of cDNA sequences that mapped to each bacterial species or higher-level taxa associated with the predicted specific metabolic genes were retrieved. The analysis was also visualized using barchart, heatmap, PCoA tree, and KEGG mapper (an internal tool based on the KEGG pathway mapping system). To confirm the focused upregulated genes, the cDNA sequences associated with those specific genes or enzymes were retrieved for designing qPCR primers. To reveal whether there was any relative change in a category, relative quantitation of gene expression was conducted. The calculation of relative changes defined by Törnqvist et al. (1985) [23] was Relative Change (υ_ref_, υ) = (υ − υ_ref_)/υ_ref_, where υ_ref_ is RA_pvc_ and υ is RA_cu_, indicating the RA of PVC downstream biofilm and the RA of the Cu downstream biofilm, respectively. Here, when the relative change is >0, 0, or <0, upregulation, no change, or downregulation occurs, respectively. Pearson correlations, multiple comparison tests, and linear analysis under PROC GLM were performed using SAS 9.4 (SAS Institute Inc., Cary, NC, USA).

### 2.6. Taxonomic Analysis and Statistical Analysis of the mRNA Sequences

MG-RAST BLAT results, which contained species information, were used to assign functional genes to specific bacteria at the genus level. Specifically, the BLAT results from two 2.4 Gbp cDNA datasets were filtered at thresholds of a maximum e-value of 1 × 10^−5^, a minimum identity cutoff of 85%, and a minimum alignment length cutoff of 80%. Then, enzyme coding sequences, including cDNA sequences, were displayed in a heat map with genus-level affiliations and gene abundance. Only those genera with more than 2 or 20 hits in the cDNA or DNA dataset, respectively, were displayed in the heat map. The annotation was based on the data compared to M5nr using a maximum e-value of 1 × 10^−5^, a minimum identity of 60%, and a minimum alignment length of 15 measured in aa for protein and bp for RNA databases. The data were normalized to values between 0 and 1.

### 2.7. RT-qPCR Validations

To validate the RNA-Seq data, reverse transcription quantitative polymerase chain reaction (RT-qPCR) assays targeting the genes associated with highly upregulated metabolic pathways like antibiotic resistance gene (*ceoB*), mycolic acids related gene (*acp*), antigen gene (85A and 85C), and ATP-binding protein gene (*kupB*), in addition to general pathogens and opportunistic pathogens, were carried out using the primers listed in Table S1. For those primers designed in this study, the sequences were retrieved from RNA-Seq libraries. For the RT-qPCR, total RNA was reverse-transcribed to cDNA using the high-capacity cDNA reverse transcription kit following the manufacture’s protocol (Thermo Fisher Scientific, Waltham, MA, USA). The qPCR reaction mixtures (20 μL) contained 10 μL 2× qPCR SYBR™ Green Master Mix (Thermo Fisher Scientific), 0.25 μM primers (final concentration), and 2 μL of template cDNA. The following cycling protocol was used with a QuantStudio 6 Flex system (Thermo Fisher Scientific): Initial cDNA treatment consisted of 50 °C for 2 min, and then 95 °C for 10 min for cDNA denaturing, 40 cycles of 15 s at 95 °C, and 1 min at 60 °C. Each cDNA sample was assayed for potential qPCR inhibitors with 10-fold dilution versus original cDNA. For each qPCR run used for validation, the threshold cycle (Ct) was normalized to the Ct of the reference genes (16S rRNA gene for *Legionella* and 23S rRNA gene for Mycobacterium) amplified from the corresponding samples. The normalized Ct is denoted as ΔCt, which can be separated as ΔCtpvc for the PVC downstream biofilm and Ctcu for the Cu downstream biofilm. The RT-qPCR fold change (2−ΔΔCT) was calculated by comparing ΔCtcu and ΔCtpvc. Here, when the fold change is >1, 1, or <1, upregulation, no change, or downregulation occurs, respectively. For pathogens present in RNA sequences that are homologous to a certain genus or species, if its qPCR signals showed positive, the organism was confirmed; otherwise, the sequences are identified as a pathogen-like organism hereinafter.

## 3. Results

### 3.1. Retrieved RNA Sequences

Overall, the meta-transcriptomic sequences totaling 95 ± 37 Mbp per library were sequenced in both directions, providing an average read length of 167 ± 17 bp. Sequence data showed only approximately 5% (5.4 ± 1.6%) of the rRNA retrieved out of the total cDNA sequences, indicating that the majority (~94%) of ribosomal sequences were removed, while mRNA was enriched efficiently.

### 3.2. Comparisons of Microbial Community Between PVC- and Cu-Influenced Biofilms

**Figure 1:**
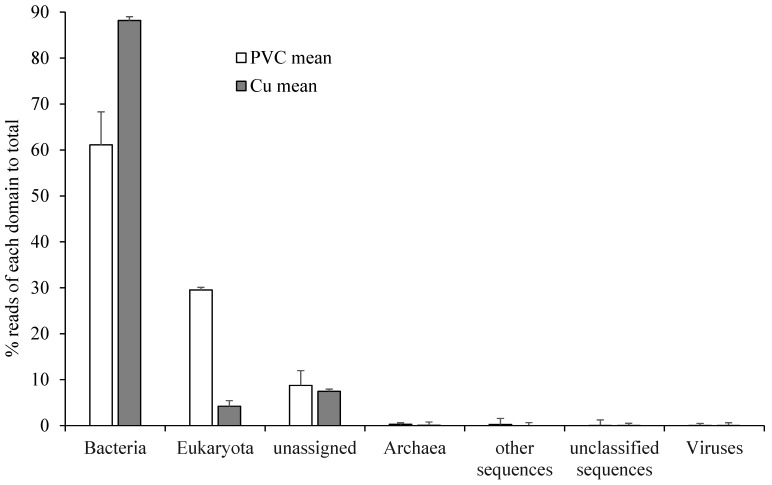
Domain distributions (total 100% for mean values) for PVC- or Cu-influenced biofilms.

**Figure 2:**
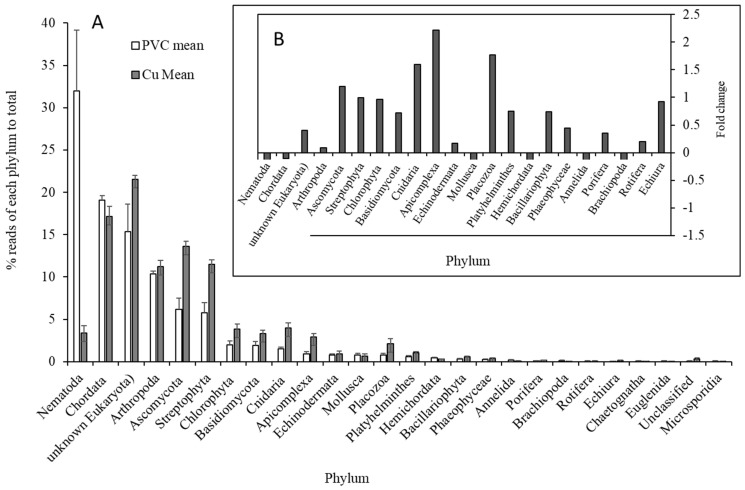
Eukaryotic phylum distribution (showed for those >0.05%, total 100% for mean values for PVC- or Cu-influenced biofilms, error bars represent one standard deviation) (**A**); insert (**B**) illustrates the relative change (fold) of phyla from PVC to Cu.

**Figure 3:**
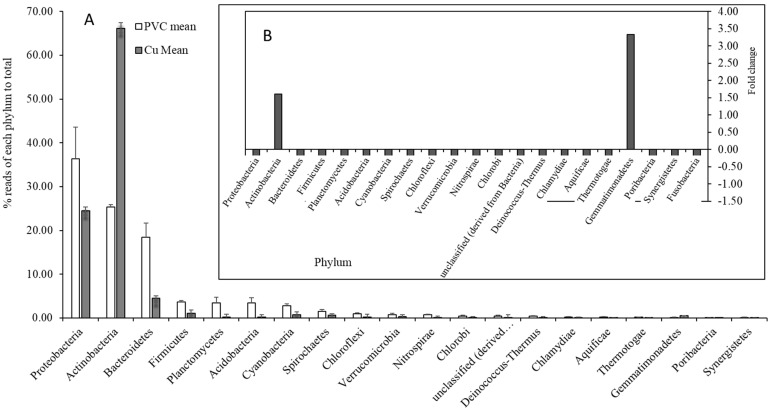
Prokaryotic phylum distribution (showed for those >0.05%, total 100% for mean values for PVC- or Cu-influenced biofilms, error bars represent standard deviations) (**A**); insert (**B**) illustrates the relative change (fold) of phyla from PVC to Cu.

The relative abundance of microbial organisms based on the functional genes showed bacterial dominance (61.1% in PVC-influenced biofilms and 88.2% in Cu-influenced biofilms), while eukaryota comprised approximately 29.5% in the PVC-influenced biofilms and 4.2% in the Cu-influenced biofilms of total sequences. Sequences from archaea and viruses accounted for less than 1% (Figure 1). Compared with PVC-influenced biofilms, only one quarter of the species occurred in Cu-influenced biofilms (α-species diversity: 444 ± 27 vs. 98 ± 23; genus: 35 vs. 23; and phylum: 21 vs. 11, respectively). With respect to abundance compared to PVC-influenced biofilms, bacteria in the Cu-influenced biofilms increased by 44% (*p* < 0.001), although the relative abundances of the majority of bacterial taxa and some eukaryota decreased (Figure 1). Among those highly decreased eukaryotic taxa, Nematoda decreased by 45% (Figure 2). Of the phyla showing a significant increase in abundance (Figure 3A), *Mycobacteriaceae* (Actinobacteria: increased by 1.61-fold increase, which were dominant in both PVC and Cu biofilms, became an absolute dominant family within Cu-influenced biofilm Actinobacteria (81.0%)). Other bacteria present were *Nocardiaceae* (increase in Actinobacteria by 12%, Figure 3B), Gemmatimonadetes (3.33-fold increase, Figure 3B), and Alphaproteobacteria (0.54-fold increase). Specifically, the majority of cDNA sequences similar to *Mycobacterium* spp. (identity 84%), mainly *Mycobacterium abscessus*, which was confirmed using qPCR, also increased (Table 1 and Table 2), and amoeba-resistant bacteria (ARB) (*Rhodococcus*, *Rhodobacter*, and *Rhodopseudomonas,* etc.) increased by 0.23–2.77-fold in Cu-influenced biofilms compared to PVC-influenced biofilms (Table 2). The mRNA functional gene sequencing results revealed that the microbial diversity and the abundance of the majority of bacterial taxa were significantly lower, but *Mycobacterium* spp. and *Rhodobacteraceae* were significantly more abundant in Cu-influenced biofilms compared to PVC-influenced biofilms. This is consistent with previous observations made from the same experiments using Sanger sequencing chemistry targeting the 16S rRNA gene [12].

### 3.3. Functional Waterborne Pathogens

In general, genera that include waterborne-pathogen-like sequences (*Acanthamoeba*, *Campylobacter*, *Cryptosporidium*, *Escherichia*, *Giardia*, *Legionella*, *Mycobacterium*, *Naegleria*, *Pseudomonas*, *Salmonella*, and *V. vermiformis*, etc.) were present in all biofilm RNA samples. The presence of various opportunistic pathogens (*Vermamoeba*, *Legionella*, *Mycobacterium,* and *Campylobacter*) was confirmed using RT-qPCR (Table 1). The confirmation from the RT-qPCR results also indicated a decrease in *Vermamoeba* and *Legionella* and an increase in *Mycobacterium* in quantity in Cu-influenced biofilms. The presence of pathogen-like sequences indicated that there were diverse viable pathogen-like organisms living in drinking water biofilms (Table 1).

For the eukaryotic taxa, nematodes accounted for 32% of the eukaryotic relative abundance and were largely *Caenorhabditis*-like OTUs (average identity: 82%) in PVC-influenced biofilms. However, nematodes were lower (3.4%) in Cu-influenced biofilms compared to PVC-influenced biofilms (11%)) (Figure 2A). Additionally, parasite-like eukaryotes (*Brugia*: identity 83%; *Loa* (filarial nematode): identity 77%) were lower in Cu-influenced biofilms compared to PVC-influenced biofilms as well. However, most other eukaryotic-like sequences were higher in Cu-influenced biofilms compared to PVC-influenced biofilms, indicating there was less of an impact of corroded Cu on eukaryotes than prokaryotes (Figure 2A).

### 3.4. Significant Regulated Functional Genes

**Figure 4:**
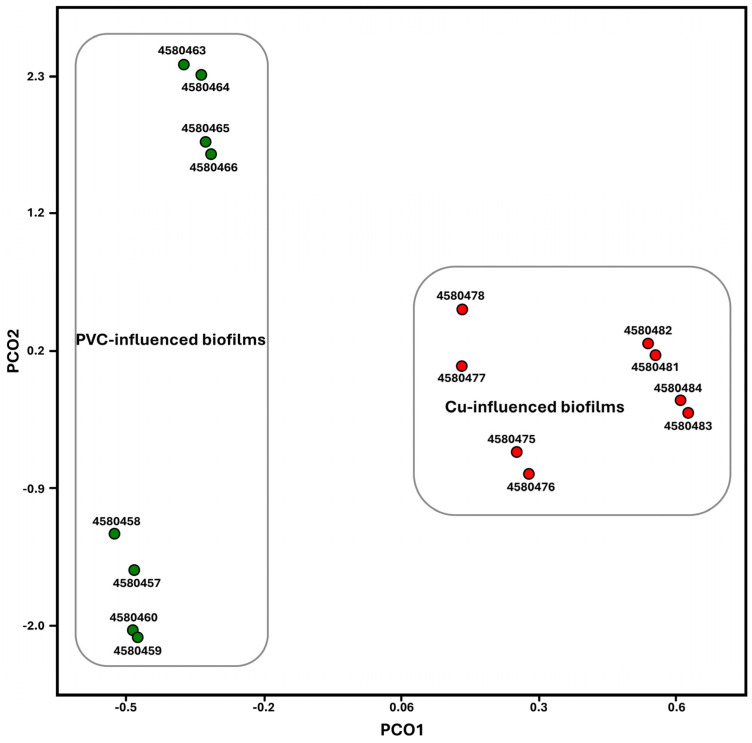
Principal component analysis of sequence library comparison for PVC- and CU-influenced biofilm samples using MG-RAST ordination plot. An analysis of similarities identified significant differences (*p* < 0.05) in PVC- and Cu-biofilm samples.

**Figure 5:**
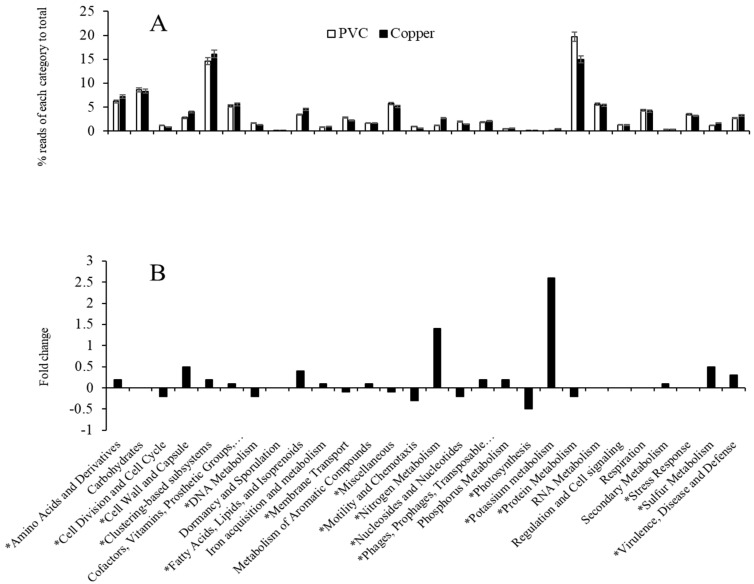
Relative abundance (**A**) and fold change (Cu vs. PVC) (**B**) of the major functional categories. * indicates that these categories are statistically significant with *p* ≤ 0.05.

The sequence libraries from PVC- and Cu-influenced biofilms were significantly clustered into two groups (Figure 4). The two groups were separated by the influenced PP materials: PVC- vs. Cu-influenced biofilms. Compared to PVC-influenced biofilms, Cu-influenced biofilms showed significantly upregulated metabolisms. They included categories of amino acids and derivatives, cell wall and capsule functions, membrane-associated fatty acid biosynthesis, lipids and isoprenoids, potassium, sulfur, and phosphorus, as well as virulence, disease, and defense. Those that were significantly downregulated were associated with DNA and protein metabolism, membrane transport, motility and chemotaxis, nucleosides and nucleotides, and stress response pathways (Figure 5).

**Figure 6:**
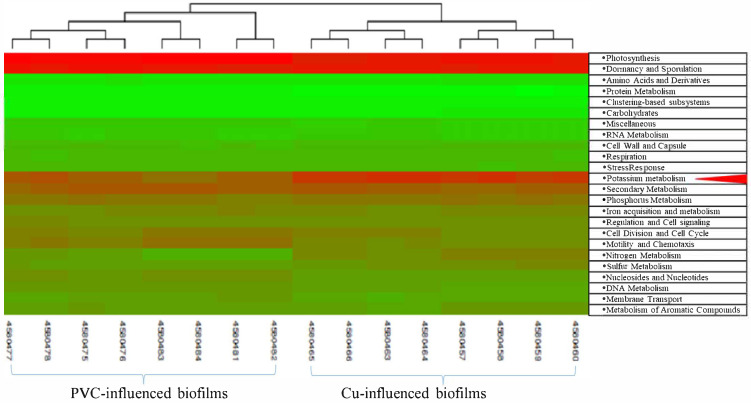
Metabolic gene heatmap analysis for PVC- and Cu-influenced biofilm sequence libraries. The color spectra show gene expressions from downregulation (green) to upregulation (red). The red arrow indicates significantly upregulated potassium metabolism in Cu-biofilm samples.

Based on the gene expression heat map (Figure 6 and Table 3), potassium metabolism appeared to be most impacted in Cu- vs. PVC-influenced biofilms. The major impact was on potassium homeostasis in Cu-influenced biofilms. The potassium-transporting ATPase A chain was upregulated, while various key genes where downregulated, including cell-membrane-associated binding genes (ATP/GTP-binding site motif A, binding-protein-dependent transport system inner membrane: FKBP-type peptidyl-prolyl cis-trans isomerase), uptake (potassium uptake protein: TrkH; potassium voltage-gated channel subfamily, TrkAH-N:Potassium), and efflux (glutathione-regulated potassium–efflux system ATP-binding protein: KefA, KefC, KdpE, Kup system) (Table 3). Other important functions associated with potassium metabolism were also impacted, such as stress (universal stress protein family (USP): upregulated), defense (adhesion, resistance to antibiotics and toxic compounds: upregulated), transport (potassium homeostasis: upregulated), translocation (Cu translocating: upregulated), nitrate and nitrite ammonification (nitrate/nitrite transporter: upregulated), ammonia assimilation (upregulated), and oxidative/reductive-associated function (heat shock: upregulated) (Table 3). The commonly impacted genes in both PVC- and Cu-influenced biofilms were those related to oxidative stress and antioxidant metabolic pathways. Some of the other potassium-associated genes include antigen 85-A and -C precursors, USP, nitrate and nitrite ammonification, ammonia assimilation, WhiB and WhiB-type regulatory proteins, thiosulfate sulfurtransferase, heme-degrading monooxygenase and ferredoxin, aspartate aminotransferase and glutamate dehydrogenases, 3-oxoacyl-[acyl-carrier protein] reductase and synthase, glutathione reductase, ferredoxin–sulfite reductase, and iron–sulfur cluster assembly protein *suf* genes.

Among those significantly upregulated categories, cell wall and capsule functional categories were explored and it was found that the mycobacteria major cell wall component, mycolic acids, was upregulated (0.94-fold increase) in Cu-influenced biofilms. This unique response was not found in the other Gram-negative and -positive cell wall components and capsular and extracellular polysaccharides. This upregulation resulted in the absolute dominance of *Mycobacteriaceae* (Actinobacteria: 1.61-fold increase) in Cu-influenced biofilms.

### 3.5. Validation of Some Functional Genes

To validate some of the highly significant mRNA changes observed, several primers targeting those transcripts were developed (Table S1) and RT-qPCR against total RNA was conducted. Generally, the RT-qPCR signals were consistent with sequenced transcripts (Table 1 and Table 4). For example, *M. abscessus* (MA) was highly dominant in the Cu-influenced biofilm samples; the genes associated with its antibiotic-like *ceoB*, an antibiotic-resistant gene with the N terminus of the K+ uptake regulatory protein (TrkA), and especially the MAC acp, a pathway involved in the synthesis and processing of mycolic acids, were upregulated. Conversely, *kupB*, a *L. pneumophila* K(+) transporter, was downregulated, which was consistent with the decrease in *L. pneumophila* detected using qPCR from Cu-influenced biofilm (Table 4).

## 4. Discussion

### 4.1. The Impact on the Functional Microbial Community

In previously described DNA clone sequence libraries, we showed that upstream Cu pipe materials significantly reduced downstream microbial diversity, resulting in the significant overgrowth of *Mycobacterium* spp. (38% of the total abundance, identity 99%) and *Pseudomonadota* (Alphaproteobacteria) (10% abundance) [12]. The fact that the two dominant groups were outgrown in Cu-influenced biofilms explained the higher bacterially expressed genes in Cu-influenced biofilms compared to PVC-influenced biofilms. Using deep-sequenced paired RNA data, this study showed similar results and further revealed the decrease in community diversity (α-diversity) and abundance of the same major bacterial taxa (Table 2). Additionally, regarding eukaryotes, there was a decrease in nematoda and an approximately one-fold decease in *Vermamoeba* but an increase in the abundance of amoeba-resistant bacteria and *Mycobacterium* spp. The consistent result of community structures in both libraries of DNA and RNA demonstrated that the tubing biofilm microbiota were largely viable, with deep sequencing analyses revealing more diversity and higher resolution. For example, not only was there higher diversity in opportunistic pathogens of *Legionella* spp., *Campylobacter* spp., and amoebae, etc., but it also identified other potentially important eukaryotic groups like nematodes, which are known to be transport hosts of human pathogens [24]. In relation to the identification of potential pathogens in sequences, only a few opportunistic pathogens commonly found in drinking water systems were confirmed. Other pathogen-like organisms may exist in drinking water at levels below the detection limit or could simply be similar organisms. In normal drinking water, there may not be a significant risk. However, under specific conditions—such as in experiments or in kitchen accessories with room temperature, slow flow, or stagnant water—rapidly growing biofilms can serve as matrices for their accumulation.

The analysis of functional gene sequences showed that biofilms harbored a diverse array of potential enteric and saprozoic pathogens in the tubing biofilms downstream of both Cu and PVC pipe materials. The shedding of the biofilm might cause more risks than previously estimated by culture-based methods, which may miss these apparently viable organisms. The frequent detection of rapidly growing mycobacteria (RGM), *M. abscessus* and *M. chelonae*, is also consistent with their isolation from potable water supplies and, in particular, where Cu piping is used, such as in health care settings and associated infections reported therein [25,26,27]. Other important opportunistic pathogens detected include *Legionella* and *Pseudomonas*, along with their biofilm host amoeba (*Vermamoeba vermiformis*; identity: 97%) [1]. Overall, the impact of Cu on these opportunistic pathogens had the same effect that it had on the general microbial communities. For example, Cu-influenced biofilms had significantly reduced populations of *Acanthamoeba*, *Campylobacter*, *Cryptosporidium, Giardia,* and *Legionella*, and caused only minor (not significant) changes to *Escherichia*, *Pseudomonas*, and *V. vermiformis*, but promoted *M. abscessus* (identity: 95%) and *M. chelonae* (identity: 99%) (Table 1 and Table 2). Interestingly, *V. vermiformis* is more often associated with legionellosis in warm/hot-water PP [28] systems that are generally made from Cu pipes, but the current study only focused on the cold-water PP. Though viruses were a very minor part of the community (Figure 1), one member identified was *Mimivirus* (identity: 72%), which is an amoebal pathogen and has been associated with both community- and hospital-acquired pneumonia [29,30].

Nematodes were previously reported to occur abundantly in drinking water and biofilm samples and were considered as hosts of pathogens in addition to causing esthetic concerns [18,31]. In this study, we found that nematodes were the dominant eukaryotic taxa in PVC-influenced biofilms and were most impacted by Cu (Figure 2). Interestingly, nematodes have long been used as bioindicators of soil and aquatic ecosystem stress and more recently through the use of molecular barcoding approaches [32]. The American Society for Testing and Materials (ASTM) published a standardized guide for soil toxicity testing with *Caenorhabditis elegans* [33], and Cu ions released from Cu plumbing materials could be lethal to nematoda. For example, the acute toxic effects of Cu on *C. elegans* (LC_50_) for reproduction and movement were estimated to be 2.04 and 2.06 mg L^−1^, respectively [34], while a significant reduction in the growth, reproduction, and lifespan of *C. elegans* occurred after chronic exposure to 0.64–6.37 mg L^−1^ Cu from 10 to 20 days [35]. In a study on Cu release using a Cu test-loop pilot system with Cincinnati drinking water, released Cu concentrations from corroding Cu pipes over time ranged between approximately 1.0 and 1.5 mg L^−1^ and up to 2.0 mg L^−1^ at pH 7.2 during a ~200 day test period [36]. Hence, Cu released from drinking water plumbing materials/biofilm is likely to be toxic to nematoda, and this group of metazoans could also be useful indicators in drinking water systems [37]. Likely, the copper released from PP, to a certain level, is toxic to bacteria. A limitation of this study is the absence of measurements for released Cu concentrations in the tubing, which affects the accuracy of describing Cu’s impact on the microbial community. From a review of drinking water Cu levels and the *Legionella* colonization of premise plumbing systems in Italy, a threshold of Cu above 0.05 mg L^−1^ was associated with minimal legionellae detections [38]. When investigating the efficacy of Cu treatment at an Ohio hospital in the United States, the higher concentration of 0.27 mg L^−1^ Cu ions (with 0.03 mg L^−1^ Ag) appeared to provide effective control of legionellae [39].

### 4.2. Impact of Copper on Microbial Organism Functions

Cu is an integral part of many important enzymes involved in a number of vital biological processes. It functions as a cofactor in enzymes that catalyze a wide variety of redox reactions due to its ability to cycle between two oxidation states, Cu^+^ and Cu^2+^ [40,41]. However, as discussed above, Cu^+^ is also toxic to cells, principally by disrupting Fe–S centers and participating in harmful Fenton reactions [42,43]. The excessive uptake of Cu may cause the disruption of microbial Cu ion homeostasis, which enables both the precise metalation of diverse cuproproteins and the control of different metal levels [44]. On the other hand, the long-term selective pressure of raised Cu concentrations may have unintended consequences in drinking water systems. Bacteria have evolved to have Cu-handling processes, which include Cu ion export, Cu ion sequestration, multi-Cu oxidases, the regulation of Cu tolerance gene expression, and Cu tolerance as a determinant of virulence, to maintain a cytoplasmic milieu that is devoid of free Cu [40]. In this study, we explored those above-mentioned Cu-handling processes that microbiota have evolved based on both the observed significant up- or downregulated genes between the PVC- and Cu-influenced biofilm communities (Figure 4, Figure 5 and Figure 6). Although there was a limitation in transcript counts due to the absence of distribution models for validating differential expressions, some key findings were validated using the RT-qPCR method, which is considered a reliable validation technique [45].

Regarding to Cu ion export, we observed that the most upregulated gene in response to Cu was potassium-transporting ATPase (3.29-fold increase), which is also associated with Cu^+^ export [46]. The exporters upregulated the genes included potassium-transporting ATPase, K^+^-carboxylase beta chain, and universal stress protein family, which could increase the plasma membrane transport of Gram-positive bacteria [47] or transport in the inner membranes to the periplasmic space in Gram-negative bacteria [48]. The protein Cu chaperones or their cognate P-type ATPases (P1B-ATPases) are thought to facilitate the delivery of Cu to these exporters through integral membrane proteins that couple ATP hydrolysis to metal cation transport [40]. The P-type ATPases (potassium-associated Cu^+^-ATPases) were also highly expressed (1.8-fold increase) and they are essential to maintain Cu^+^ homeostasis [49]. In contrast, other potassium-associated binding metabolisms like ATP/GTP-binding site motif A, binding-protein-dependent transport systems’ inner membrane (FKBP-type peptidyl-prolyl cis-trans isomerase), potassium uptake protein (TrkH), potassium voltage-gated channel subfamily, TrkA-N potassium, and glutathione-regulated potassium–efflux system ATP-binding protein (KefA, KefC, KdpE, Kup system) [50,51] were inhibited. The result of downregulated expression (validated by qPCR) of the *kup* gene, which encodes K^+^ transporters of *L. pneumophila*, indicated the suppressive impact of Cu on the Kup protein. This resulted in *Legionella* being in agreement with the response reflected in cDNA sequences (Table 3). Hence, the response was specific to Cu, minimizing the impacts of the potassium-associated binding processes. The response of bacteria to Cu ions in this study was also consistent with the previous findings in a culture of a Cu-resistant strain of *Mycobacterium smegmatis* under a stress of 19.2 mg L^−1^ Cu ions [52]. The previous major finding was the upregulated Cu-translocating P-type ATPase, which indicated that the Cu-resistant strain had a five-fold-higher resistance to Cu sulfate than its parental strain.

Another mechanism for Cu tolerance is that of Cu ion sequestration. For example, our finding of hemin uptake and utilization system upregulation (Table 3) may implicate Cu^+^ sequestration. As observed with siderophores produced in *Yersinia* spp. (yersiniabactin or Ybt) and a similar finding in *E. coli* isolates, these siderophores are also capable of binding Cu^++^ to reduce Cu ion toxicity [53]. Additionally, our observed upregulated proteins like WhiB and WhiB-type (proteins for bacterial cyanide reduction) [54] were possibly also associated with Cu sequestration. A recent review of siderophore-mediated antimicrobial resistance (AMR) also points to another way that Cu could influence AMR in drinking water systems [55].

The general oxidative stress responses observed with the upregulation of glutaredoxins and sulfur metabolism (Figure 6 and Table 3) could be an additional mechanism of preventing the generation of toxic cuprous ions. Bacterial multi-Cu oxidases that are known to confer Cu tolerance include CueO in *E. coli* and *S. enterica* Typhimurium and MmcO in *M. tuberculosis* [56,57]. In a study on *L. pneumophila*’s transcriptional response to chlorine treatment, the result showed a chlorine-induced stress response in a *L. pneumophila* lens that was related to the induction of cellular protective processes [58]. The enhanced expression of cellular antioxidant proteins, stress proteins, or transcriptional regulators may reflect an attempt by the bacteria to maintain cellular homeostasis. A notable example of these proteins is the universal stress protein (UspA), which becomes highly abundant in cells that have ceased growing and when they are subjected to a wide variety of environmental stressors [59]. The resistance to oxidative stress could also have gone through the glutamate and aspartate metabolic pathways, as associated with cytotoxic end product formation in *Porphyromonas gingivalis* [60]. Since iron–sulfur proteins are involved in diverse and vital physiological processes ranging from energy metabolism to DNA replication and repair [61,62], the upregulation of iron–sulfur proteins could prevent a broad impact on multiple physiological functions in cells due to the inactivation of iron–sulfur proteins by Cu.

A key finding of the current study was the distinct effect of upstream Cu exposure on *Mycobacterium* compared to non-*Mycobacterium* species. Specifically, genes associated with the synthesis of major cell wall components and enzymes, such as mycolic acids, acyl carrier protein, and linoleoyl-CoA desaturase, were found to be upregulated in mycobacteria (Figure 6 and Table 3). In contrast, there was no observed upregulation for genes involved in the cell wall components of Gram-negative and other Gram-positive bacteria, nor for capsular and extracellular polysaccharides. This suggests that the strengthening of the *Mycobacterium* cell wall’s major components may be a crucial adaptation, allowing mycobacteria to dominate over other bacteria when exposed to Cu stress. Cu’s toxic effects on microorganisms primarily involve disrupting the integrity of the plasma membrane [63]. Mycolic acids in *Mycobacterium* species are crucial for growth and survival, not only by conferring resistance to chemical damage and dehydration, but also by inhibiting the efficacy of hydrophobic antibiotics [64]. Furthermore, these acids facilitate the bacterium’s growth inside macrophages/amoebae, shielding it from the host immune response. For example, the biosynthesis of mycolate is vital for the survival and pathogenicity of *M. tuberculosis* during prolonged exposure to Cu. *Mycobacterium* antigen proteins (antigen 85, which was significantly upregulated) are known to exhibit mycolyl transferase activity, likely playing a role in cell wall synthesis [65].

## 5. Conclusions

The released Cu ions from the upstream biocorrosion of Cu pipe materials caused significant toxicity to downstream biofilm microbiota. Generally, Cu decreased microbial diversity, the abundance of the majority of bacterial taxa, and the overall relative abundance of eukaryotes, particularly nematodes (largely *Caenorhabditis*-like OTUs). Within drinking water biofilms, diverse viable pathogen-like organisms coexisted; the majority were negatively impacted by Cu exposure. Conversely, *Mycobacterium* spp., primarily *M. abscessus*, and amoeba-resistant bacteria (ARB) such as *Rhodococcus*, *Rhodobacter*, and *Rhodopseudomonas* were more abundant in Cu-influenced biofilms compared to PVC-influenced biofilms. Two significant findings emerged from the microbial transcriptomic response to Cu toxicity: potassium metabolism and cell wall components and enzymes in *Mycobacterium* spp. The genes associated with potassium transport, particularly the upregulated potassium-transporting ATPase, facilitate the export of excess Cu ions. Conversely, genes linked to potassium-binding metabolisms were downregulated, presumably to avoid the uptake of Cu ions. The upregulated genes associated with the synthesis of major cell wall components and enzymes were mainly for the synthesis of mycolic acids, that provide antibiotic-resistance, reduced Cu-induced ROS and limits membrane permeability of Cu ions. These adaptations likely confer resistance to Cu-induced damage and dehydration, providing a selective advantage for mycobacteria to dominate in PP biofilms. Additionally, other upregulated genes may play roles in Cu ion sequestration, multi-Cu oxidation, and the regulation of Cu tolerance. The result provides new insights into how copper pipe materials influence the development and composition of biofilms in premise plumbing, either by inhibiting the growth of many microbes or by contributing to the resistance of some microbial adaptations. Understanding how copper pipes influence biofilm development can help in designing plumbing systems that minimize harmful microbial growth, leading to safer drinking water. Although the findings are limited in laboratory conditions, the information underscores the need for further research using real-world biofilm samples. Such research can lead to more practical and applicable solutions in the field of water safety and plumbing.

## Figures and Tables

**Table 1:** Pathogen-related mRNA sequences from the downstream tubing biofilms associated with PVC and copper pipes.

Genus	Sequence Fold Change ^1^	***: *p* ≤ 0.001	PVC Mean	StDev	Cu Mean	StDev	Reference Species Mainly Hit	RT-qPCR Fold Change ^2^	**: *p* ≤ 0.01
*Acanthamoeba*	−0.86	***	0.54	0.259	0.075	0.035	*A. castellanii*, *A. culbertsoni*, *A. healyi*, *A. polyphaga*		
*Campylobacter*	−0.60	***	0.03	0.01	0.01	0.00	*C. jejuni*, *C. hominis*, *C. Concisus*, *C. Curvus*, *C. fetus*, *C. lari*, *C. rectus*, *C. coli*, *C. showae*		
*Cryptosporidium*	−0.67	***	0.06	0.01	0.02	0.01	*C. parvum*, *C. hominis*, *C. muris*		
*Escherichia*	−0.29		0.11	0.05	0.08	0.06	*E. coli*		
*Giardia*	−0.69	***	0.01	0.00	0.00	0.00	*G. intestinalis*		
*Vermamoeba*	−0.38		0.32	0.22	0.20	0.21	*V. vermiformis*	0.81	**
*Legionella*	−0.91	***	0.55	0.11	0.05	0.02	*L. pneumophila*, *L. longbeachae*, *L. drancourtii*	0.91	**
*Mimivirus*	−0.86	***	0.00	0.00	0.00	0.00	*A. polyphaga mimivirus*		
*Mycobacterium*	3.97	***	10.25	3.38	50.94	4.81	*M. abscessus*, *M. avium*, *M. bovis*, *M. chelonae*, *M. gilvum*, *M. marinum*, *M. smegmatis*, *C. tuberculosis*, *C. smegmatis*, *M. gilvum*, *C. marinum, M. parascrofulaceum*, *M. microti,*	3.06	**
*Naegleria*	−0.76	***	0.17	0.04	0.04	0.02	*N. gruberi*, *N. fowleri*		
*Pseudomonas*	−0.23		1.05	0.53	0.80	0.45	*P. Fluorescens*, *P. aeruginosa*,		
*Salmonella*	−0.30	***	0.03	0.01	0.02	0.01	*S. enterica*, *S. bongori*		

^1^: Defined as the ratio of the difference of mean relative abundance between PVC- and Cu-influenced biofilms to the former mean relative abundance, wherein, when the fold change is >0, 0, or <0, upregulation, no change, or downregulation occurs, respectively. ^2^: Calculated as 2^(mean ΔCtpvc − mean ΔCtCu)/mean ΔCtpvc)^, where ΔCt_pvc_ indicates the normalized qPCR cycle threshold (Ct) for PVC downstream biofilm and ΔCt_cu_ is the normalized Ct of the Cu downstream biofilm. Here, when the fold change is >1, 1, or <1, upregulation, no change, or downregulation occurs, respectively.

**Table 2:** Bacteria with increased abundance in copper-influenced tubing biofilms.

Phylum	Class/Order/Family/Species	Fold Change	*: *p* ≤ 0.05, **: *p* ≤ 0.01, ***: *p* ≤ 0.001	PVC Mean	PVC StDev	Cu Mean	Cu StDev
*Actinomycetota* (*Actinobacteria*)	*Mycobacteriaceae*	0.09	***	74.34	1.20	80.98	0.63
	*M. abscessus*	0.03	***	39.13	0.42	40.38	0.73
	*M. bovis*	0.10	**	3.18	0.19	3.51	0.07
	*M. microti*	0.22	***	1.53	0.13	1.87	0.07
	*M. smegmatis*	0.06	**	6.16	0.13	6.56	0.25
	*M. tuberculosis*	0.08	**	4.50	0.15	4.88	0.13
	*M. vanbaalenii*	0.16	***	4.02	0.27	4.66	0.20
*Pseudomonadota* (*Proteobacteria*)		0.54	***	48.98	0.98	75.48	2.70
	*Rhodobacterales*	1.87	***	8.77	0.58	25.20	2.79
	*Rhodobacteraceae*	0.23	***	77.05	1.16	94.77	0.86
	*Rhodobacter* (sp. SW2)	2.77	***	12.35	0.69	46.54	1.97
	*Sphingomonadales*	0.79	***	11.10	0.62	19.88	4.95
*Bacteroidota*	*Flavobacteriia*	1.55	***	17.67	1.83	45.14	5.81
*Bacteroidota*	*Sphingobacteriia*	0.55	*	13.71	1.07	21.30	7.01

**Table 3:** Bacterial functional gene transcriptomic changes in copper-influenced tubing biofilms.

1st Category	2nd-Level Category	Fold Change (Total (Cu%-uPVC%))/Total uPVC%	*: *p* ≤ 0.05, **: *p* ≤ 0.01, ***: *p* ≤ 0.001	Major Genes Upregulated	Major Genes Downregulated
Potassium-Associated Metabolism	Potassium homeostasis	3.29	***	Potassium-transporting ATPase: A (6.5-fold), B (3.2-fold), and C (2.5-fold) chain	ATP/GTP-binding site motif A, binding-protein-dependent transport systems’ inner membrane: FKBP-type peptidyl-prolyl cis-trans isomerase; potassium uptake protein: TrkH; potassium voltage-gated channel subfamily; TrkA-N: potassium; glutathione-regulated potassium–efflux system ATP-binding protein: KefA, KefC, KdpE, Kup system
	CBSS-83332.1.peg.3803 (antigen proteins)	2.81	***	Propionyl-CoA activated by K+ carboxylase beta chain (0.5), probable polyketide synthase (0.6)	Antigen 85-A and -C precursor (85A and 85C)
	Universal stress protein family	1.80	***	Universal stress protein family: the universal stress protein (USP) domain is a superfamily of conserved genes that can be found in bacteria, archaea, fungi, protozoa, and plant proteins	
	CBSS-196620.1.peg.2477 (1–5%)	1.75	***	Copper-translocating P-type ATPase	Delta-1-pyrroline-5-carboxylate dehydrogenase, ferrous iron transport protein B, maltose O-acetyltransferase
	Nitrate and nitrite ammonification	1.58	***	Nitrite reductase [NAD(P)H] subunits, nitrate/nitrite transporter	Respiratory nitrate reductase alpha, beta, delta, and gamma chain
	Ammonia assimilation	1.51	***	uridylyltransferase: Nucleotidyl transferase is a component of the repair pathway for single-nucleotide base excision repair. This repair mechanism begins when a single nucleotide is recognized by DNA glycosylase as incorrectly matched or has been mutated in some way (UV light, chemical mutagen, etc.), and is removed. Later, a nucleotidyl tranferase is used to fill in the gap with the correct base, using the template strand as the reference.	Glutamate synthas: This enzyme belongs to the family of oxidoreductases, specifically those acting on the CH-NH2 group of donors with NAD+ or NADP+ as acceptor. This enzyme participates in glutamate metabolism and nitrogen metabolism. It has 5 cofactors: FAD, iron, FMN, sulfur, and iron–sulfur.
	WhiB and WhiB-type regulatory proteins	1.32	**	Sporulation regulatory protein WhiD and WhiB family transcriptional regulator	
	Bacterial cyanide production and tolerance mechanisms	1.18	***	Thiosulfate sulfurtransferase	Formate dehydrogenase O alpha, beta, and gama subunit
	Heme and hemin uptake and utilization systems in Gram-positive bacteria	1.12	***	Iron-dependent repressor	Heme-degrading monoxygenase
	Allophanate hydrolase 2 and biotin carboxylase cluster	0.92	***	Allophanate hydrolase 2 subunit 1 (EC 3.5.1.54)	Biotin carboxylase
	Soluble cytochromes and functionally related electron carriers	0.76	***	Ferredoxin	Cytochrome
	Heme and hemin uptake and utilization systems in Gram-negative bacteria	0.55	*	Electron transfer flavoprotein, beta subunit	Ferric siderophore transport system, periplasmic binding protein TonB, outer membrane receptor proteins, heme iron utilization protein
	RNA modification cluster	0.43	***	Inner membrane protein translocase component YidC, long form	Inner membrane protein translocase component YidC, short form OxaI-like (YidC)
Amino Acids and Derivatives	Glutamine, glutamate, aspartate, asparagine; ammonia assimilation	0.71	***	Glutamine synthetases: plays an essential role in the metabolism of nitrogen	Aspartate aminotransferase, glutamate, and aspartate uptake in bacteria; glutamate dehydrogenases
Cell Wall and Capsule	Major component of cell wall of mycobacteria: mycolic acids, but not Gram-negative and -positive cell wall components and capsular and extracellular polysaccharides	0.94	***	Antibiotics and resistance: Acyl carrier protein, linoleoyl-CoA desaturase	3-oxoacyl-[acyl-carrier protein] reductase and synthase
Fatty Acids, Lipids, and Isoprenoids	Fatty acids (biosynthesis) and phospholipids	0.34	***	Fatty acids: membrane; phospholipids: membrane and oxidant (fab genes)	Possibly associated with disruption of plasma membrane integrity; triacylglycerols
Stress Response	Heat shock	0.37	**	Heat shock DNA cluster	Oxidative stress: glutathione reductase to reduce oxidized glutathione within cells, indicating cellular toxicity by Cu.
	Oxidative stress	0.2	***	Upregulation of oxidative stress response glutaredoxins, glutathione analogs: mycothiol and CoA-disulfide reductase	Down: some glutathione redox cycles
Sulfur Metabolism	Inorganic sulfur assimilation	0.45	***		Ferredoxin–sulfite reductase, sulfate adenylyltransferase, sulfate transporter
Virulence, Disease, and Defense	Adhesion and resistance to antibiotics and toxic compounds	0.78	***	Copper homeostasis (copper-translocating P-type ATPase, copper chaperone, copper resistance protein C precursor), BlaR1 family regulatory sensor transducer disambiguation, erythromycin resistance	Ferredoxin–sulfite reductase, sulfate- and thiosulfate-binding protein CysP
Miscellaneous	Iron–sulfur cluster assembly protein		***	Iron–sulfur cluster assembly protein SufA, B, D, R (0.28–1.92)	Cysteine desulfurase and chaperone protein HscB and HscA
Nitrogen Metabolism	Nitrate/nitrite transporter Nitrite reductase [NAD(P)H] large subunit Respiratory nitrate reductase alpha chain	0.74 0.50 −3.54	** ** ***		

**Table 4:** Validation of mycobacteria and *L. pneumophila* mRNA sequences using qPCR targeting antibiotic genes.

Primers—> Downstream of:	MAC ^1^ *ceoB*f2/r2	MAC ^1^ *ceo B*f1/r1	MAC ^2^ *acp1* F1/R1	85A ^3^ F5/R5	85C ^3^ F3/R3	LP ^4^ *kupB*
PVC: mean (Ct)	25.28	25.02	18.92	30.08	27.04	32.99
PVC: variance (Ct)	0.98	0.83	1.42	0.58	0.67	0.59
Cu: mean (Ct)	24.43	24.16	16.86	30.98	28.62	35.78
Cu: variance (Ct)	0.59	0.49	0.59	0.82	0.87	0.27
P (*t*-test, *n* = 8)	0.06	0.02	0.01	0.06	0.00	0.00
Fold change (2^∆Ct^)	1.80	1.82	4.18	0.54	0.33	0.15

^1^: MAC (*Mycobacterium avium* complex) *ceoB*: Antibiotic gene with the N terminus of the K+ uptake regulatory protein (~TrkA). ^2^: MAC *acp1*: Pathway to synthesis and processing of mycolic acids. ^3^: 85A and 85C: Antigen proteins. ^4^: LP (*Legionella pneumophila*) *kupB*: ATP-binding protein genes.

## Data Availability

The original contributions presented in this study are included in the article/Supplementary Materials. Further inquiries can be directed to the corresponding author.

## Supplementary Materials

The following supporting information can be downloaded at: https://www.mdpi.com/article/10.3390/microorganisms13071528/s1, Figure S1. The CDC reactor setup (A) consists of water tank (B), bioreactors and waste effluent line and container (C, D). The water tank (B) is fed with Cincinnati tap water at a flow rate of 11.67 mL/min (residence time of 30 min) and a baffle rotation of up to 120 rpm for 1–2 months controlled using a peristaltic pump on the top of a shelf (B). Both the CDC reactors (C) and the water tank were covered with aluminum foil to prevent light. Each reactor contains 8 coupon holders, and each reactor vessel contains 24 coupons (D). Table S1. Oligonucleotide qPCR primer sequences used to screen pathogens and sequence validation. References [66,67,68,69,70,71,72,73,74,75] are cited in the Supplementary Materials.

## References

[1] Ashbolt N.J. (2015). Environmental (saprozoic) pathogens of engineered water systems: Understanding their ecology for risk assessment and management. Pathogens.

[2] Prest E.I., Hammes F., Van Loosdrecht M.C., Vrouwenvelder J.S. (2016). Biological stability of drinking water: Controlling factors, methods, and challenges. Front. Microbiol..

[3] Galarce C., Fischer D., Díez B., Vargas I.T., Pizarro G.E. (2020). Dynamics of biocorrosion in copper pipes under actual drinking water conditions. Water.

[4] Cullom A., Spencer M.S., Williams M.D., Falkinham J.O., Brown C., Edwards M.A., Pruden A. (2023). Premise plumbing pipe materials and in-building disinfectants shape the potential for proliferation of pathogens and antibiotic resistance genes. Environ. Sci. Technol..

[5] Van der Kooij D., Veenendaal H.R., Scheffer W.J. (2005). Biofilm formation and multiplication of *Legionella* in a model warm water system with pipes of copper, stainless steel and cross-linked polyethylene. Water Res..

[6] Doğruöz N., Gungor N.D., Minnnos B., Sungur E.I., Çotuk A. (2009). Biofilm formation on copper and galvanized steel surfaces in a cooling-water system. Eur. J. Biol..

[7] Yu J., Kim D., Lee T. (2010). Microbial diversity in biofilms on water distribution pipes of different materials. Water Sci. Technol..

[8] Jang H.J., Choi Y.J., Ka J.O. (2011). Effects of diverse water pipe materials on bacterial communities and water quality in the annular reactor. J. Microbiol. Biotechnol..

[9] Morvay A., Decun M., Scurtu M., Sala C., Morar A., Sarandan M. (2011). Biofilm formation on materials commonly used in household drinking water systems. Water Sci. Technol. Water Supply.

[10] Lehtola M.J., Miettinen I.T., Lampola T., Hirvonen A., Vartiainen T., Martikainen P.J. (2005). Pipeline materials modify the effectiveness of disinfectants in drinking water distribution systems. Water Res..

[11] Buse H.Y., Lu J., Lu X., Mou X., Ashbolt N.J. (2014). Microbial diversities (16S and 18S rRNA gene pyrosequencing) and environmental pathogens within drinking water biofilms grown on the common premise plumbing materials unplasticized polyvinylchloride and copper. FEMS Microbiol. Ecol..

[12] Lu J., Buse H., Gomez-Alvarez V., Struewing I., Santo Domingo J., Ashbolt N.J. (2014). Impact of drinking water conditions and copper materials on downstream biofilm microbial communities and *Legionella pneumophila* colonization. J. Appl. Microbiol..

[13] Huang C.K., Weerasekara A., Bond P.L., Weynberg K.D., Guo J. (2021). Characterizing the premise plumbing microbiome in both water and biofilms of a 50-year-old building. Sci. Total Environ..

[14] van der Kooij D., Veenendaal H.R., Italiaander R. (2020). Corroding copper and steel exposed to intermittently flowing tap water promote biofilm formation and growth of *Legionella pneumophila*. Water Res..

[15] Buse H.Y., Ji P., Gomez-Alvarez V., Pruden A., Edwards M.A., Ashbolt N.J. (2017). Effect of temperature and colonization of *Legionella pneumophila* and *Vermamoeba vermiformis* on bacterial community composition of copper drinking water biofilms. Microb. Biotechnol..

[16] Inkinen J., Jayaprakash B., Ahonen M., Pitkänen T., Mäkinen R., Pursiainen A., Santo Domingo J.W., Salonen H., Elk M., Keinänen-Toivola M.M. (2018). Bacterial community changes in copper and PEX drinking water pipeline biofilms under extra disinfection and magnetic water treatment. J. Appl. Microbiol..

[17] Gomez-Alvarez V., Revetta R.P., Santo Domingo J.W. (2012). Metagenomic analyses of drinking water receiving different disinfection treatments. Appl. Environ. Microbiol..

[18] Buse H.Y., Lu J., Struewing I.T., Ashbolt N.J. (2013). Eukaryotic diversity in premise drinking water using 18S rDNA sequencing: Implications for health risks. Environ. Sci. Pollut. Res..

[19] Revetta R., Gomez-Alvarez V., Gerke T., Santo Domingo J., Ashbolt N. (2016). Changes in bacterial composition of biofilm in a metropolitan drinking water distribution system. J. Appl. Microbiol..

[20] Proctor C.R., Reimann M., Vriens B., Hammes F. (2018). Biofilms in shower hoses. Water Res..

[21] Qin J., Li R., Raes J., Arumugam M., Burgdorf K.S., Manichanh C., Nielsen T., Pons N., Levenez F., Yamada T. (2010). A human gut microbial gene catalogue established by metagenomic sequencing. Nature.

[22] Meyer F., Paarmann D., D’Souza M., Olson R., Glass E., Kubal M., Paczian T., Rodriguez A., Stevens R., Wilke A. (2008). The metagenomics RAST server—A public resource for the automatic phylogenetic and functional analysis of metagenomes. BMC Bioinform..

[23] Törnqvist L., Vartia P., Vartia Y.O. (1985). How should relative changes be measured?. Am. Stat..

[24] Hemmerling C., Labrosse A., Ruess L., Steinert M. (2023). *Legionella pneumophila* and free-living nematodes: Environmental co-occurrence and trophic link. Microorganisms.

[25] Chadha R., Grover M., Sharma A., Lakshmy A., Deb M., Kumar A., Mehta G. (1998). An outbreak of post-surgical wound infections due to *Mycobacterium abscessus*. Pediatr. Surg. Int..

[26] Lowry P.W., Sague C.M.B., Bland L.A., Aguero S.M., Arduino M.J., Minuth A.N., Murray R.A., Swenson J.M., Jarvis W.R. (1990). *Mycobacterium chelonae* infection among patients receiving high-flux dialysis in a hemodialysis clinic in California. J. Infect. Dis..

[27] Falkinham J.O. (2016). Current epidemiologic trends of the nontuberculous mycobacteria (NTM). Curr. Environ. Health Rep..

[28] Buse H.Y., Schoen M.E., Ashbolt N.J. (2012). Legionellae in engineered systems and use of quantitative microbial risk assessment to predict exposure. Water Res..

[29] Raoult D., Audic S., Robert C., Abergel C., Renesto P., Ogata H., La Scola B., Suzan M., Claverie J.-M. (2004). The 1.2-megabase genome sequence of *Mimivirus*. Science.

[30] La Scola B., Marrie T.J., Auffray J.-P., Raoult D. (2005). Mimivirus in pneumonia patients. Emerg. Infect. Dis..

[31] Inkinen J., Jayaprakash B., Siponen S., Hokajärvi A.-M., Pursiainen A., Ikonen J., Ryzhikov I., Täubel M., Kauppinen A., Paananen J. (2019). Active eukaryotes in drinking water distribution systems of ground and surface waterworks. Microbiome.

[32] Schenk J., Höss S., Brinke M., Kleinbölting N., Brüchner-Hüttemann H., Traunspurger W. (2020). Nematodes as bioindicators of polluted sediments using metabarcoding and microscopic taxonomy. Environ. Int..

[33] (2022). Standard Guide for Conducting Laboratory Soil Toxicity Tests with the Nematode Caenorhabditis Elegans.

[34] Boyd W.A., Williams P.L. (2003). Comparison of the sensitivity of three nematode species to copper and their utility in aquatic and soil toxicity tests. Environ. Toxicol. Chem..

[35] Harada H., Kurauchi M., Hayashi R., Eki T. (2007). Shortened lifespan of nematode *Caenorhabditis elegans* after prolonged exposure to heavy metals and detergents. Ecotoxicol. Environ. Saf..

[36] Lytle D.A., Liggett J. (2016). Impact of water quality on chlorine demand of corroding copper. Water Res..

[37] van Lieverloo J.H.M., van der Kooij D., Hoogenboezem W., Bitton G. (2002). Invertebrates and protozoa (free-living) in drinking water distribution systems. Encyclopedia of Environmental Microbiology.

[38] Borella P., Guerrieri E., Marchesi I., Bondi M., Messi P. (2005). Water ecology of *Legionella* and protozoan: Environmental and public health perspectives. Biotechnol. Annu. Rev..

[39] Lin Y.-s.E., Vidic R.D., Stout J.E., Yu V.L. (2002). Negative effect of high pH on biocidal efficacy of copper and silver ions in controlling *Legionella pneumophila*. Appl. Environ. Microbiol..

[40] Ladomersky E., Petris M.J. (2015). Copper tolerance and virulence in bacteria. Metallomics.

[41] Gaetke L.M., Chow C.K. (2003). Copper toxicity, oxidative stress, and antioxidant nutrients. Toxicology.

[42] Macomber L., Imlay J.A. (2009). The iron-sulfur clusters of dehydratases are primary intracellular targets of copper toxicity. Proc. Natl. Acad. Sci. USA.

[43] Dupont C.L., Grass G., Rensing C.J.M. (2011). Copper toxicity and the origin of bacterial resistance—New insights and applications. Metallomics.

[44] Quintana J., Novoa-Aponte L., Argüello J.M. (2017). Copper homeostasis networks in the bacterium *Pseudomonas aeruginosa*. J. Biol. Chem..

[45] Everaert C., Luypaert M., Maag J.L., Cheng Q.X., Dinger M.E., Hellemans J., Mestdagh P. (2017). Benchmarking of RNA-sequencing analysis workflows using whole-transcriptome RT-qPCR expression data. Sci. Rep..

[46] Stautz J., Hellmich Y., Fuss M.F., Silberberg J.M., Devlin J.R., Stockbridge R.B., Hänelt I. (2021). Molecular mechanisms for bacterial potassium homeostasis. J. Mol. Biol..

[47] Solioz M., Abicht H.K., Mermod M., Mancini S. (2010). Response of Gram-positive bacteria to copper stress. JBIC J. Biol. Inorg. Chem..

[48] Argüello J.M., Eren E., González-Guerrero M. (2007). The structure and function of heavy metal transport P 1B-ATPases. Biometals.

[49] González-Guerrero M., Argüello J.M. (2008). Mechanism of Cu+-transporting ATPases: Soluble Cu+ chaperones directly transfer Cu+ to transmembrane transport sites. Proc. Natl. Acad. Sci. USA.

[50] Jung K., Altendorf K. (2002). Towards an understanding of the molecular mechanisms of stimulus perception and signal transduction by the KdpD/KdpE system of *Escherichia coli*. J. Mol. Microbiol. Biotechnol..

[51] Cholo M.C., van Rensburg E.J., Osman A.G., Anderson R. (2015). Expression of the genes encoding the *Trk* and *Kdp* potassium transport systems of *Mycobacterium tuberculosis* during growth in vitro. BioMed Res. Int..

[52] Chen Y., Yang F., Sun Z., Wang Q., Mi K., Deng H. (2015). Proteomic analysis of drug-resistant mycobacteria: Co-evolution of copper and INH resistance. PLoS ONE.

[53] Chaturvedi K.S., Hung C.S., Crowley J.R., Stapleton A.E., Henderson J.P. (2012). The siderophore yersiniabactin binds copper to protect pathogens during infection. Nat. Chem. Biol..

[54] Bush M.J., Tschowri N., Schlimpert S., Flärdh K., Buttner M.J. (2015). c-di-GMP signalling and the regulation of developmental transitions in streptomycetes. Nat. Rev. Microbiol..

[55] Khan F.Z., Palmer K., Dillon N. (2024). Siderophores mediate antibiotic resistance. Nat. Microbiol..

[56] Roberts S.A., Weichsel A., Grass G., Thakali K., Hazzard J.T., Tollin G., Rensing C., Montfort W.R. (2002). Crystal structure and electron transfer kinetics of CueO, a multicopper oxidase required for copper homeostasis in *Escherichia coli*. Proc. Natl. Acad. Sci. USA.

[57] Rowland J.L., Niederweis M. (2012). Resistance mechanisms of *Mycobacterium tuberculosis* against phagosomal copper overload. Tuberculosis.

[58] Bodet C., Sahr T., Dupuy M., Buchrieser C., Héchard Y. (2012). *Legionella pneumophila* transcriptional response to chlorine treatment. Water Res..

[59] O’Connor A., McClean S. (2017). The role of universal stress proteins in bacterial infections. Curr. Med. Chem..

[60] Takahashi N., Sato T., Yamada T. (2000). Metabolic pathways for cytotoxic end product formation from glutamate-and aspartate-containing peptides by *Porphyromonas gingivalis*. J. Bacteriol..

[61] Johnson D.C., Dean D.R., Smith A.D., Johnson M.K. (2005). Structure, function, and formation of biological iron-sulfur clusters. Annu. Rev. Biochem..

[62] Paul V.D., Lill R. (2015). Biogenesis of cytosolic and nuclear iron–sulfur proteins and their role in genome stability. Biochim. Et Biophys. Acta-Mol. Cell Res..

[63] Avery S.V., Howlett N.G., Radice S. (1996). Copper toxicity towards *Saccharomyces cerevisiae*: Dependence on plasma membrane fatty acid composition. Appl. Environ. Microbiol..

[64] Portevin D., de Sousa-D’Auria C., Houssin C., Grimaldi C., Chami M., Daffé M., Guilhot C. (2004). A polyketide synthase catalyzes the last condensation step of mycolic acid biosynthesis in mycobacteria and related organisms. Proc. Natl. Acad. Sci. USA.

[65] Armitige L.Y., Jagannath C., Wanger A.R., Norris S.J. (2000). Disruption of the genes encoding antigen 85A and antigen 85B of *Mycobacterium tuberculosis* H37Rv: Effect on growth in culture and in macrophages. Infect. Immun..

[66] Linton D., Owen R.J., Stanley J. (1996). Rapid identification by PCR of the genus Campylobacter and of five Campylobacter species enteropathogenic for man and animals. Res. Microbiol..

[67] Lund M., Nordentoft S., Pedersen K., Madsen M. (2004). Detection of *Campylobacter* spp. in chicken fecal samples by real-time PCR. J. Clin. Microbiol..

[68] González-Escalona N., Hammack T.S., Russell M., Jacobson A.P., De Jesús A.J., Brown E.W., Lampel K.A. (2009). Detection of live *Salmonella* sp. cells in produce by a TaqMan-based quantitative reverse transcriptase real-time PCR targeting invA mRNA. Appl. Environ. Microbiol..

[69] Bruijnesteijn van Coppenraet E.S., Lindeboom J.A., Prins J.M., Peeters M.F., Claas E.C.J., Kuijper E.J. (2004). Real-time PCR assay using fine-needle aspirates and tissue biopsy specimens for rapid diagnosis of mycobacterial lymphadenitis in children. J. Clin. Microbiol..

[70] Riviere D., Szczebara F.M., Berjeaud J.M., Frere J., Hechard Y. (2006). Development of a real-time PCR assay for quantification of *Acanthamoeba* trophozoites and cysts. J. Microbiol. Methods.

[71] Kuiper M.W., Valster R.M., Wullings B.A., Boonstra H., Smidt H., van der Kooij D. (2006). Quantitative detection of the free-living amoeba *Hartmannella vermiformis* in surface water by using real-time PCR. Appl. Environ. Microbiol..

[72] Schroeder J.M., Booton G.C., Hay J., Niszl I.A., Seal D.V., Markus M.B., Fuerst P.A., Byers T.J. (2001). Use of subgenic 18S ribosomal DNA PCR and sequencing for genes and genotype identification of acanthamoebae from human with keratitis and sewage sludge. J. Clin. Microbiol..

[73] Hadfield S.J., Robinson G., Elwin K., Chalmers R.M. (2011). Detection and differentiation of *Cryptosporidium* spp. in human clinical samples by use of realtime PCR. J. Clin. Microbiol..

[74] Guy R.A., Payment P., Krull U.J., Horgen P.A. (2003). Real-time PCR for quantification of Giardia and Cryptosporidium in environmental water samples and sewage. Appl. Environ. Microbiol..

[75] Qvarnstrom Y., Visvesvara G.S., Sriram R., da Silva A.J. (2006). Multiplex real-time PCR assay for simultaneous detection of *Acanthamoeba* spp.; *Balamuthia mandrillaris*, and *Naegleria fowleri*. J. Clin. Microbiol..

