# Supplementary material for: Meta-Transcriptomic Response to Copper Corrosion in Drinking Water Biofilms
Source: Microorganisms. 2025 Jun 30;13(7):1528. doi: 10.3390/microorganisms13071528 (PMC12300335; doi:10.3390/microorganisms13071528)
Supplement: Supplementary file 1 [file microorganisms-13-01528-s001.zip › microorganisms-3681237-supplementary.pdf]

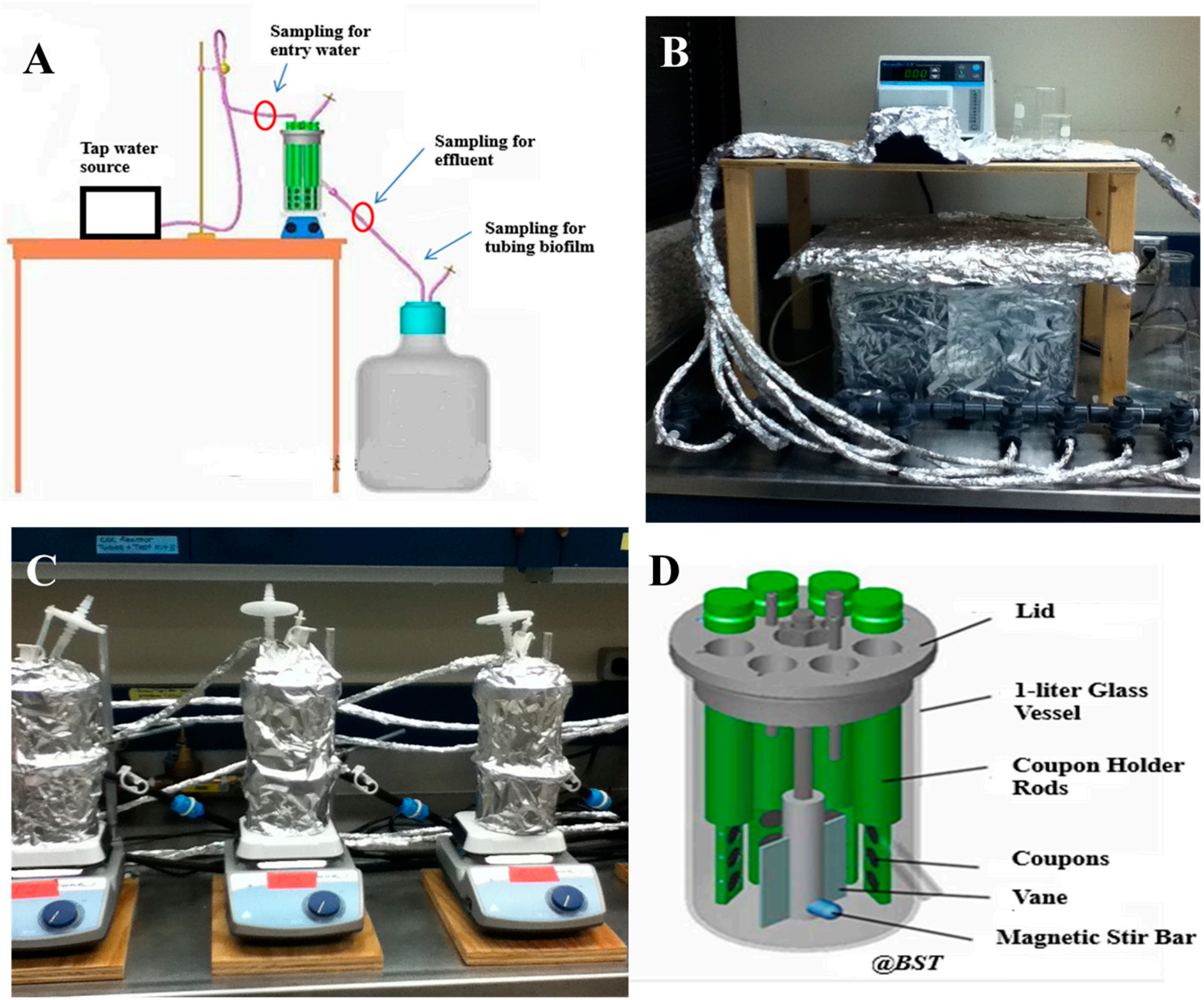
A
Sampling for entry water
Sampling for effluent
Tap water source
Sampling for tubing biofilm
B
C
D
Lid
1-liter Glass Vessel
Coupon Holder Rods
Coupons
Vane
Magnetic Stir Bar
@BST

Figure S1. The CDC reactor setup (A) consists of water tank (B), bioreactors and waste effluent line and container (C-D). The water tank (B) is fed with Cincinnati tap water at a flow rate of 11.67mL/min (residence time of 30 minutes) and a baffle rotation of up to 120 rpm for 1-2 months controlled using a peristaltic pump on the top of a shelf (B). Both the CDC reactors (C) and the water tank were covered with aluminum foil to prevent light. Each reactor contains 8 coupon holders, and each reactor vessel contains 24 coupons (D)

Table S1. Oligonucleotide qPCR primer sequences used to screen pathogens and sequence validation

Target	Oligo name	Sequences (5'-3')	Tm (°C)	Detection limit (CE per reaction)	Reference
Campylobacter spp. (qPCR)	CampF2	CACGTGCTACAATGGCATAT	58	1	Lund et al. 2004
	CampR2	GGCTTCATGCTCTCGAGTT			
	CampP2	FAM-CAGAGAACAATCCGAACTGGGACA-TAMRA			
Campylobacter spp. (PCR)	CampF	GGA TGA CAC TTT TCG GAG C	60	2	Linton, 1996
	CampR	CAT TGT AGC ACG TGT GTC			
Legionella spp. (qPCR)	Leg F1c	TAGTGGAATTTCCGGTGTA	50	2	This study
	Leg R1c	CCAACAGCTAGTTGACATC			
	Leg-probe	6Fam-CGGCTACCTGGCCTAATACTGA-Tamra			
Salmonella spp. (qPCR)	invA_176F	CAACGTTTCCTGCGGTACTGT	60	4	Gonzalez-Escalona, N., et al. 2009
	invA-Tx_208	FAM-CTCTTTCGTCTGGCATTATCGATCAGTACCA-TAMRA			
	invA_291R	CCCGAACGTGGCGATAATT			
Mycobacterium spp. (qPCR)	23SmycoF	GGG GTGTGGTGTTTGAG	62	1	Bruijnesteijn van Coppenraet, 2004
	23SmycoR	CTCCCACGTCCTTCATC			
	23SmycoProbe	6-carboxyfluorescein-TGGATAGTGGTTGCGAGCATC-BHQ1			
Trk system potassium uptake protein CeoB [*M. abscessus*, NC_010397]	MA-ceoBf1	GGCGATCATCGACAAGGAA	62	NA	This study
	MA-ceoBr1	GTAAGAGAACGTCGCGATCAA			
	MA-ceoBf2	GTGCGAGTAGTGGTGATGG			
	MA-ceoBr2	CGGCTTCCTTGTCGATGAT			
mycolic acid synthesis: STEAROYL-ACP DESATURASE	MA-acp1 F1	TCG AAG AGG TCA CCG GTA T			
	MA-acp1 R1	ATC TTC ACG CCG TAC TTG TC			
Potassium homeostasis antigen 85A and 85C *[M. abscessus]*	MA-85A F5	TGG TCA GTC CAG CTT CTA CA			
	MA-85A R5	CCC TTG TTC GCA GCA AGA TA			
	MA-85C F3	TCA ACG GCT GGG ATA TCA AC			
	MA-85C R3	CGA ATA CCA GTC GGT GTA GAA G			
K^+ transporter	Lp-kupBf	TCGTGCTGATAATGATGG	60		
	Lp-kupBr	GCCTCACCACCAGTCA			

Acanthamoeba spp. (qPCR)	TaqAcF1	CGACCAGCGATTAGGAGACG	60	2	Riviere et al. 2006
	TaqAcR1	CCGACGCCAAGGACGAC			
	TaqAcP1	FAM-TGAATACAAAACACCACCATCGGCGC-TAMRA			
Vermamoeba vermiformis (qPCR)	Hv1227F	TTA CGA GGT CAG GAC ACT GT	56	2	Kuiper et al. 2006
	Hv1728R	GAC CAT CCG GAG TTC TCG			
Acanthamoeba spp. (PCR)	JDP1	GGCCCAGATCGTTTACCGTGA A	60	2	Schroeder et al. 2001
	JDP2	TCTCACAAGCTGCTAGGGAGTCA			
Cryptosporidium spp. (qPCR)	CRU18Sf	GAG GTA GTG ACA AGA AAT AAC AAT ACA GG	60	2	Hadfield et al 2011
	r	CTG CTT TAA GCA CTC TAA TTT TCT CAA AG			
	P	6FAM-TAC GAG CTT TTT AAC TGC AAC AA-BHQ			
	JVA18S f	ATG ACG GGT AAC GGG GAA T	60	2	Hill et al. 2007
	r	CCA ATT ACA AAA CCA AAA AGT CC			
	p	CGC GCC TGC TGC CTT CCT TAG ATG			
Giardia spp. qPCR	β-Giardin P241 F	CATCCGCGAGGAGGTCAA	60	2	Guy, 2003
	R	GCAGCCATGGTGTCGATCT			
	P	FAM/AAGTCCGCCGACAACATGTACCTAACGA/BHQ-1			
Naegleria fowleri	NaegIF192	GTG CTG AAA CCT AGC TAT TGT AAC TCA GT	63	1	Qvarnstrom et al. 2006
	NaegIR344	CAC TAG AAA AAG CAA ACC TGA AAG G			
	NfowlP	HEX-AT AGC AAT ATA TTC AGG GGA GCT GGG C-BHQ1			

References

66 Linton, D., ; Owen, R. J.; Stanley, J. (1996) Rapid identification by PCR of the genus Campylobacter and of five Campylobacter species enteropathogenic for man and animals." *Res. Microbiol*. 147 707-718.

67 Lund M.; Nordentoft S.; Pedersen K'; Madsen M. (2004) Detection of *Campylobacter* spp. in chicken fecal samples by real-time PCR, *J Clin Microbiol* **42**:.42:5125-32.

68 Gonzalez-Escalona N., et al. (2009) Detection of live Salmonella sp. cells in produce by a TaqMan-based quantitative reverse transcriptase real-time PCR targeting invA mRNA. *Appl Environ Microbiol* **75**:3714–3720.

69 Bruijnesteijn van Coppenraet, E.S., Lindeboom, J.A., Prins, J.M., Peeters, M.F., E. Claas, C.J. and Kuijper, E.J. (2004) Real-time PCR assay using fine-needle aspirates and tissue biopsy specimens for rapid diagnosis of mycobacterial lymphadenitis in children. *J Clin Microbiol* **42**:2644

70 Riviere, D., Szczebara, F.M., Berjeaud, J.M., Frere, J. and Hechard, Y. (2006) Development of a real-time PCR assay for quantification of Acanthamoeba trophozoites and cysts. J *Microbiol Methods* **64**, 78–83.

71 Kuiper, M. W.; Valster, R. M.; Wullings, B. A.; Boonstra, H.; Smidt, H.; van der Kooij, D. (2006). Quantitative detection of the free-living amoeba Hartmannella vermiformis in surface water by using real-time PCR. *Appl. Environ. Microbiol. 72*:5750-5756.

72 Schroeder, J.M., Booton, G.C., Hay, J., Niszl, I.A., Seal, D.V., Markus, M.B., Fuerst, P.A. and Byers, T.J. (2001) Use of subgenic 18S ribosomal DNA PCR and sequencing for genes and genotype identification of acanthamoebae from human with keratitis and sewage sludge. *J Clin Microbiol* **39***:*1903–1911.

73 Hadfield, S.J., Robinson, G, Elwin, K., Chalmers, R.M. (2011) Detection and differentiation of *Cryptosporidium* spp. in human clinical samples by use of realtime PCR. *J Clin Microbiol* **49**: 918–924.

74 Guy, R. A.; Payment, P.; Krull, U. J.; Horgen, P. A. (2003). Real-time PCR for quantification of Giardia and Cryptosporidium in environmental water samples and sewage. *Appl. Environ. Microbiol. 69:*5178-5185.

75 Qvarnstrom, Y., Visvesvara, G.S., Sriram, R. and da Silva, A. J. (2006). Multiplex real-time PCR assay for simultaneous detection of *Acanthamoeba* spp., *Balamuthia mandrillaris*, and *Naegleria fowleri*. *J Clin Microbiol* **44**, 3589-3595.
